# Associations among knee muscle strength, structural damage, and pain and mobility in individuals with osteoarthritis and symptomatic meniscal tear

**DOI:** 10.1186/s12891-018-2182-8

**Published:** 2018-07-27

**Authors:** Brittney A. Luc-Harkey, Clare E. Safran-Norton, Lisa A. Mandl, Jeffrey N. Katz, Elena Losina

**Affiliations:** 1 Orthopaedic and Arthritis Center for Outcomes Research, Department of Orthopedic Surgery, Brigham and Women’s Hospital, 60 Fenwood Road, Suite 5016, Boston, MA 02115 USA; 2 Rehabilitation Services, Brigham and Women’s Hospital, Boston, MA USA; 3 Weill Cornell Medicine, Hospital for Special Surgery, New York, NY USA

**Keywords:** Quadriceps, Hamstrings, Timed up and go, KOOS; activities of daily living

## Abstract

**Background:**

Sufficient lower extremity muscle strength is necessary for performing functional tasks, and individuals with knee osteoarthritis demonstrate thigh muscle weakness compared to controls. It has been suggested that lower muscle strength is associated with a variety of clinical features including pain, mobility, and functional performance, yet these relationships have not been fully explored in patients with symptomatic meniscal tear in addition to knee osteoarthritis. Our purpose was to evaluate the associations of quadriceps and hamstrings muscle strength with structural damage and clinical features in individuals with knee osteoarthritis and symptomatic meniscal tear.

**Methods:**

We performed a cross-sectional study using baseline data from the Meniscal Tear in Osteoarthritis Research (MeTeOR) trial. We assessed structural damage using Kellgren-Lawrence grade and the magnetic resonance imaging osteoarthritis knee score (MOAKS) for cartilage damage. We used the Knee Injury and Osteoarthritis Outcomes Score (KOOS) to evaluate pain, symptoms, and activities of daily living (ADL), and the Timed Up and Go (TUG) test to assess mobility. We assessed quadriceps and hamstrings strength using a hand-held dynamometer and classified each into quartiles (Q). We used Chi square tests to evaluate the association between strength and structural damage; and separate analysis of covariance models to establish the association between pain, symptoms, ADL and mobility with strength, after adjusting for demographic characteristics (age, sex and BMI) and structural damage.

**Results:**

Two hundred fifty two participants were evaluated. For quadriceps strength, subjects in the strongest quartile scored 14 and 13 points higher on the KOOS Pain and ADL subscales, respectively, and completed the TUG two seconds faster than subjects in the weakest quartile. For hamstrings strength, subjects in the strongest quartile scored 13 and 14 points higher on the KOOS pain and ADL subscales, respectively, and completed the TUG two seconds faster than subjects in the weakest quartile. Strength was not associated with structural damage.

**Conclusions:**

Greater quadriceps and hamstrings muscle strength was associated with less pain, less difficulty completing activities of daily living, and better mobility. These relationships should be evaluated longitudinally.

## Background

Knee osteoarthritis is a leading cause of functional limitation in older adults [1, 2]. Functional limitation is manifest as reductions in both self-reported difficulty performing activities of daily living and in objectively quantified mobility [3–5]. Identifying modifiable factors that contribute to pain, difficulty performing activities of daily living, and mobility is necessary to inform efficient therapeutic regimens that effectively reduce functional limitation in individuals with knee osteoarthritis.

Performing functional tasks requires sufficient lower extremity muscle strength [6]. Individuals with symptomatic knee osteoarthritis demonstrate deficits in quadriceps and hamstrings strength when compared to healthy matched controls [7–10]. It has been suggested that lower quadriceps and hamstrings strength contribute to a variety of clinical features, including poorer patient-reported function [11, 12], worse physical performance [13–15] and disease progression [16]. The relationship between lower extremity muscle strength and clinical features may be confounded, however by radiographic disease severity [17] in addition to demographic characteristics such as sex [18–20] and body mass index (BMI) [18]. As muscle strength is modifiable, understanding the associations between quadriceps and hamstrings strength and a broad set of clinical features could help to determine efficacious treatment targets.

Previous studies assessing the associations between quadriceps and hamstrings muscle strength and clinical features of knee osteoarthritis have quantified muscle strength using instrumentation only available in research laboratories [13–15, 21, 22], precluding their applicability in the clinical setting. Hand-held dynamometers are small, portable devices that allow for the measurement of muscle strength in clinical settings. Strong, positive associations (i.e., Pearson correlation coefficient range = 0.72–0.85) between the assessment of quadriceps and hamstrings muscle strength using a hand-held dynamometer and an isokinetic dynamometer have been reported [23]. Additionally, hand-held dynamometers demonstrate excellent inter-rater and inter-session reliability [24]. Limited research demonstrates hip abductor strength, assessed using a hand-held dynamometer associates with physical function in patients with knee osteoarthritis [25]. Therefore, our purpose was to evaluate the associations among quadriceps and hamstrings strength measured with a hand-held dynamometer and 1) structural damage quantified via radiograph and MRI, 2) patient-reported pain, symptoms and difficulty performing activities of daily living quantified via the Knee Injury and Osteoarthritis Outcomes Score (KOOS), and 3) performance of mobility tasks quantified via the Timed Up and Go (TUG) test in individuals with symptomatic meniscal tear and osteoarthritis. We hypothesized that less quadriceps and hamstrings strength is associated with greater structural damage (i.e. radiographic severity and depth and size of cartilage damage), greater patient-reported limitations, and poorer mobility.

## Methods

### Study design

This cross-sectional study utilized baseline data from the Meniscal Tear in Osteoarthritis Research (MeTeOR) trial (NCT00597012). The MeTeOR trial is a multi-center randomized controlled trial comparing arthroscopic partial meniscectomy plus physical therapy versus physical therapy alone for the treatment of symptomatic meniscal tear. Details of the MeTeOR trial have been published previously [26, 27]. At the time of enrollment, participants underwent a baseline imaging assessment and a testing session that included the assessment of patient-reported outcomes, the Timed Up and Go (TUG) test, quadriceps strength and hamstrings strength. All study procedures were approved by the Partners HealthCare Human Research Committee, and all participants provided written consent prior to participation.

### Participants

Participants were recruited into the MeTeOR Trial from seven academic referral centers between June 2008 and August 2011 [26, 27]. Briefly, eligible patients of the participating surgeons across each center were identified, screened for eligibility, and referred to research coordinators if interested in participating. Full trial procedures have been published previously [27]. Participants were 45 years or older, and had an MRI of the knee with evidence of a meniscal tear that extended to the meniscal surface. Included participants had baseline imaging evidence of osteoarthritic changes as determined by either MRI evidence of osteophytes or full-thickness cartilage defect, or plain radiographic evidence of osteophytes or joint space narrowing. Exclusion criteria comprised a chronically locked knee, inflammatory arthritis, injection of corticosteroids or hyaluronic acid agents within the past four weeks, contraindications to surgery or physical therapy, bilateral symptomatic meniscal tears, and prior surgery on the index knee. Participants receiving an injection within the previous 4 weeks were excluded as evidence supports short-term improvement in osteoarthritis symptoms following injections [28], and this could obscure treatment effects [27]. Participants with radiographic Kellgren-Lawrence (K-L) grade 4 were also excluded as total knee arthroplasty is more appropriate than arthroscopic partial meniscectomy in this setting [27]. For this study, we analyzed data from each participant’s index knee, which we defined as the knee that prompted the participant to seek care. The MeTeOR Trial was powered to detect a 10-point difference in the WOMAC function scale at 6 months, which was the principal trial outcomes measure. Adopting a Type I error rate of 5% and power of 80%, the target sample size was set at 340 participants. A total of 351 participants were enrolled into the MeTeOR Trial.

### Quadriceps strength

**Fig. 1:**
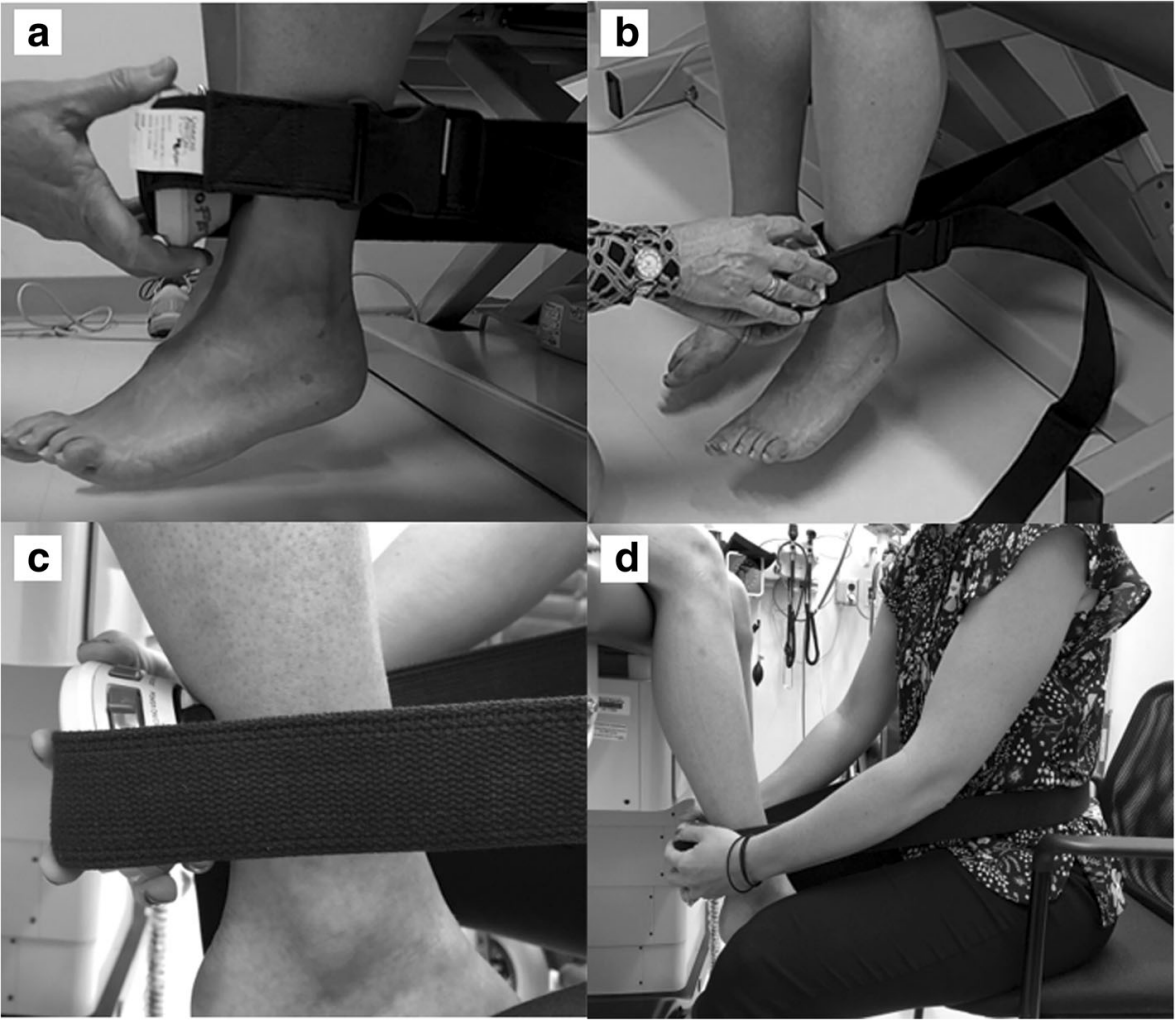
Muscle Strength Assessment. Legend: For the assessment of quadriceps strength, **a**) the hand-held dynamometer was positioned on the anterior aspect of the distal tibia, just superior to the malleoli, and **b**) participants were seated on an examination table with their knees flexed to 60° and their feet off the ground. An inelastic strap was secured around the treatment table under the participant, and was used to maintain the position of the hand-held dynamometer and the knee angle during each testing trial. For the assessment of hamstring strength, **c**) the hand-held dynamometer was positioned on the posterior aspect of the lower leg, just superior to the malleoli, and **d**) participants were seated on an examination table with their knees flexed to 60° and their feet off the ground. An inelastic strap was secured around the waist of the assessor who was seated directly in front of the participant. The strap was used to maintain the position of the hand-held dynamometer and the knee angle during each testing trial

Maximal voluntary isometric quadriceps muscle strength was assessed using a hand-held dynamometer (Fig. 1; MicroFET 2; Hoggan Scientific, LLC, Salt Lake City, UT). Participants were seated on an examination table with their knees flexed to 60° and their feet off the ground. The hand-held dynamometer was positioned on the anterior aspect of the distal tibia, just superior to the malleoli. An inelastic strap was secured around the treatment table under the participant, and was used to maintain the position of the hand-held dynamometer and the knee angle during each testing trial [29, 30]. Participants grasped the examination table with their hands for stabilization, and participants were instructed to extend their knee “as hard as possible” into the hand-held dynamometer. Participants continued to exert force into the hand-held dynamometer for 4 s, and the maximum force across the trial was recorded. Three testing trials were completed, and we normalized the average force (Newtons [N]) across the three trials to body mass (N/kg) [19].

### Hamstrings strength

Maximal voluntary isometric hamstrings muscle strength was assessed with participants seated on an examination table with their knees flexed to 60° and their feet off the ground (Fig. 1). The hand-held dynamometer was positioned on the posterior aspect of the lower leg, just superior to the malleoli [29]. An inelastic strap was secured around the waist of the assessor who was seated directly in front of the participant. The strap was used to maintain the position of the hand-held dynamometer and the knee angle during each testing trial. Participants grasped the examination table with their hands for stabilization, and participants were instructed to flex their knee “as hard as possible” into the hand-held dynamometer. Participants continued to exert force into the hand-held dynamometer for 4 s, and the maximum force across the trial was recorded. Three testing trials were completed, and we normalized the average force (Newtons [N]) across the three trials to body mass (N/kg) [19]. For both quadriceps and hamstrings strength, we categorized the normalized strength values into quartiles (Q) for analysis, with the lowest quartile indicative of the poorest strength.

### Structural damage

Structural damage was determined based upon radiographic K-L grade and the size and thickness of cartilage damage on MRI. Radiographic K-L grade was categorized as 0) no radiographic features of osteoarthritis, 1) doubtful joint space narrowing and questionable osteophyte formation, 2) possible joint space narrowing and definite osteophyte formation, and 3) multiple osteophytes and joint space narrowing < 50% [26]. The MRIs were re-read centrally by a single experienced musculoskeletal radiologist, and the size and depth of cartilage damage was classified per the MRI OA Knee Score (MOAKS) criteria. The MOAKS is a semi-quantitative assessment of structural features consistent with knee OA [31]. The MOAKS divides the articular and sub-spinous regions of the knee into 14 sub-regions to quantify the size of cartilage loss (% of surface area in each sub-region) and the depth of cartilage damage (% of full thickness loss in each sub-region). The 14 sub-regions include the patella (medial patella; lateral patella), the femur (medial trochlea; lateral trochlea; medial central femur; lateral central femur; medial posterior femur; lateral posterior femur), and the tibia (anterior medial tibia; middle medial tibia; posterior medial tibia; anterior lateral tibia; middle lateral tibia; posterior lateral tibia) [31]. The size of cartilage damage was categorized as 0) none, 1) < 10%, 2) 10–75%, and 3) > 75%. The depth of cartilage damage was categorized as 0) none, 1) < 10%, 2) 10–75%, and 3) > 75%. All 14 sub-regions were assessed, and we used the maximum score for the size of cartilage damage and for the depth of cartilage damage for analysis [31].

### Patient-reported pain and functional status

We assessed baseline patient-reported outcomes using the pain, symptoms and function in activities of daily living (ADL) subscales of the Knee injury and Osteoarthritis Outcomes Score (KOOS) which is a valid and reliable instrument [32]. Within each subscale, scores from each item were summed and divided by the maximum possible score and multiplied by 100 to create a normalized score ranging from 0 to 100. Lower scores indicate greater pain, greater severity of symptoms, and greater difficulty in performing activities of daily living [32]. A difference of 8 points on each KOOS subscale between quartiles of quadriceps and hamstrings muscle strength represents a clinically meaningful difference in patient reported pain, symptoms and difficulty performing ADL [32].

### Mobility

We assessed mobility using the Timed Up and Go (TUG) test [33]. Participants began seated in a chair, and were instructed to rise from the chair without the use of their arms for support, walk 3 m at a self-selected comfortable speed, and return to the seated position. A stopwatch was used to record the time interval from when the participant was instructed to begin the test until s/he returned to the seated position. The TUG test demonstrates excellent test-retest reliability in individuals with knee osteoarthritis [33]. Time to complete the test was recorded in seconds; a longer time to complete the TUG indicates poorer mobility. A difference of 1.14 s on the TUG test between quadriceps and hamstrings strength quartiles represents a clinically meaningful difference in mobility [33].

### Statistical analysis

We used means and percentages to describe the sample characteristics. We used separate Chi square tests to determine differences in structural damage (K-L grade and the maximum MOAKS score for size and depth of cartilage damage) across quartiles of quadriceps strength and hamstrings strength. Next, we analyzed the associations between the clinical features (KOOS pain score, KOOS symptoms score, KOOS ADL score, and time to complete the TUG test) and quartiles of quadriceps strength and hamstrings strength using separate analysis of covariance models. We applied a threshold of *P* < 0.05 to determine statistical significance after adjusting for demographic characteristics (age, sex and BMI) and structural damage (K-L grade and the maximum MOAKS score for size and depth of cartilage damage). All analyses were performed using SAS 9.4 statistical software (SAS Institute Inc., Cary, NC).

## Results

### Sample characteristics

Of the 351 participants who were enrolled into the MeTeOR trial 252 had complete quadriceps and hamstrings strength and clinical features data. Therefore, our sample was comprised of 252 participants. The baseline characteristics for study participants, are presented in Table 1. The mean age for the entire cohort was 58 years (standard deviation [SD] 7; range = 45–87), BMI was 30 kg/m^2^ (SD 6; range = 19–51), and 57% were female. With respect to structural damage, 9% had K-L Grade 0, 21% had K-L Grade 1, 42% had K-L Grade 2, and 28% had K-L Grade 3. Thirty-one percent received a maximum MOAKS cartilage damage depth score of 0, 23% received a 1, 37% received a 2, and 9% received a 3. Two percent of participants received a maximum MOAKS cartilage damage size score of 0, 5% received a 1, 54% received a 2, and 39% received a 3. The mean scores for the KOOS pain, symptoms and ADL subscales was 54 (SD 16; range = 8–97), 45 (SD 15; range = 10–100) and 64 (SD 18; range = 12–100), respectively, and the mean time to complete the TUG was 10 (SD 4; range = 1–30) seconds. The mean quadriceps strength on the index limb was 1.89 N/kg (SD 0.89; range = 0.18–5.03) and the mean hamstrings strength was 1.39 N/kg (SD 0.66; range = 0.18–3.67).

**Table 1:** Participant characteristics by strength quartile

	Quartile 1 (weakest)	Quartile 2	Quartile 3	Quartile 4 (strongest)	*P* value
	Quadriceps Strength				
Age	56 (6)	56 (7)	61 (7)	60 (8)	0.889
Sex					0.021
Male	20%	40%	45%	64%	
Female	80%	60%	55%	36%	
BMI	33 (7)	31 (6)	28 (4)	27 (4)	0.061
MOAKS Cartilage Damage Depth Score					0.169
0	27%	41%	20%	36%	
1	22%	22%	24%	25%	
2	44%	29%	41%	34%	
3	7%	8%	15%	5%	
MOAKS Cartilage Damage Size Score					0.011
0	2%	2%	0%	6%	
1	0%	6%	5%	9%	
2	59%	63%	41%	52%	
3	39%	29%	54%	33%	
K-L Grade					0.002
0	12%	13%	9%	5%	
1	12%	27%	12%	33%	
2	39%	49%	39%	39%	
3	37%	11%	39%	23%	
Quadriceps Strength (N/kg)	0.86 (0.28)	1.47 (0.13)	2.02 (0.20)	3.11 (0.57)	< 0.001
KOOS Pain	44 (16)	53 (14)	55 (13)	62 (15)	< 0.001
KOOS Symptoms	43 (15)	47 (15)	43 (15)	47 (13)	0.226
KOOS ADL	53 (19)	60 (17)	67 (14)	72 (16)	< 0.001
TUG Test (s)	12 (5)	10 (4)	9 (3)	9 (2)	0.008
	Hamstrings Strength				
Age	57 (7)	58 (7)	59 (8)	60 (7)	0.627
Sex					0.007
Male	26%	33%	51%	63%	
Female	74%	67%	49%	37%	
BMI	32 (7)	31 (6)	28 (5)	27 (4)	0.081
MOAKS Cartilage Damage Depth Score					0.840
0	35%	30%	30%	28%	
1	21%	16%	29%	28%	
2	36%	43%	33%	35%	
3	8%	11%	8%	8%	
MOAKS Cartilage Damage Size Score					0.544
0	2%	2%	2%	5%	
1	2%	8%	6%	5%	
2	62%	54%	46%	52%	
3	35%	36%	46%	38%	
K-L Grade					0.358
0	11%	14%	9%	3%	
1	15%	16%	29%	25%	
2	47%	44%	33%	42%	
3	27%	25%	29%	30%	
Hamstrings Strength (N/kg)	0.64 (0.17)	1.10 (0.12)	1.57 (0.15)	2.30(0.41)	< 0.001
KOOS Pain	46 (16)	51 (14)	55 (14)	63 (14)	< 0.001
KOOS Symptoms	44 (15)	45 (16)	44 (15)	47 (14)	0.925
KOOS ADL	55 (18)	60 (18)	66 (16)	74 (14)	< 0.001
TUG Test (s)	12 (5)	10 (3)	9 (3)	9 (2)	0.005

Continuous data presented as mean (standard deviation); categorical data presented percentage; MOAKS = MRI Knee OA Score; KOOS = knee injury and osteoarthritis outcomes score; BMI = body mass index; K-L = Kellgren-Lawrence; N = newtons; ADL = activities of daily living; TUG = Timed Up and Go; *p*-value corresponds to the difference in each outcome measure across quartiles of muscle strength

### Quadriceps Strength Associations with Structural Damage, Patient Reported Pain and Functional Status, and Mobility

The distribution of K-L grades significantly differed across quartiles of quadriceps strength (Table 1; *p* = 0.002). The distribution of the maximum MOAKS score for cartilage damage size significantly differed across quartiles of quadriceps strength (Table 1; *p* = 0.011). We did not find that the distribution of the maximum MOAKS score for cartilage damage depth was different across quadriceps strength quartiles (Table 1; *p* = 0.169).

**Fig. 2:**
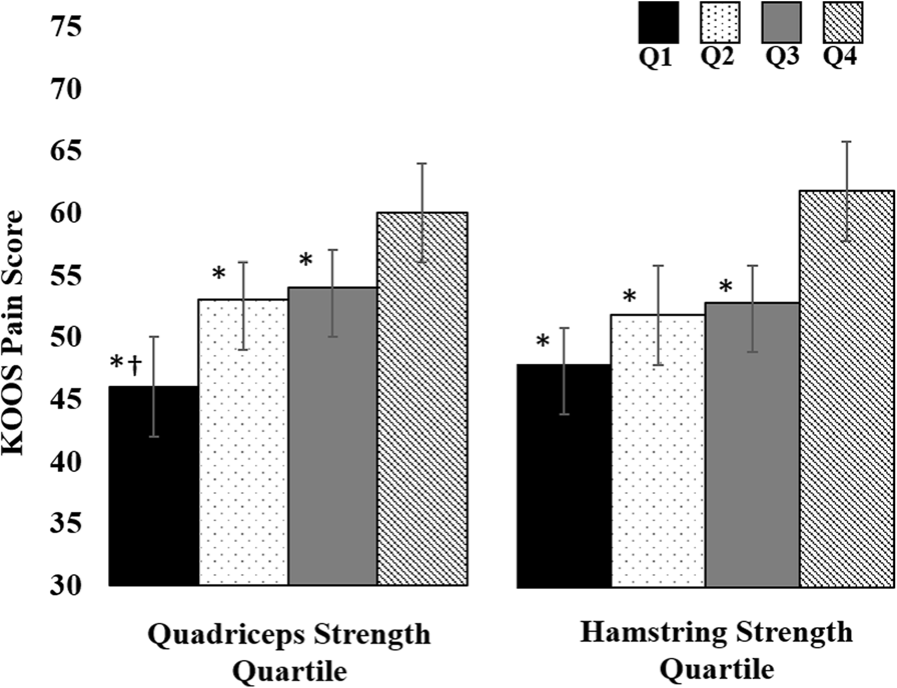
KOOS Pain Scores by Quartile of Muscle Strength. Legend: Data presented as mean Knee injury and Osteoarthritis Outcomes Score (KOOS) pain scores across each quartile (Q) of muscle strength adjusting for structural damage, age, sex and BMI. * indicates significantly less than quartile 4; † indicates significantly less than quartile 2 and quartile 3

**Fig. 3:**
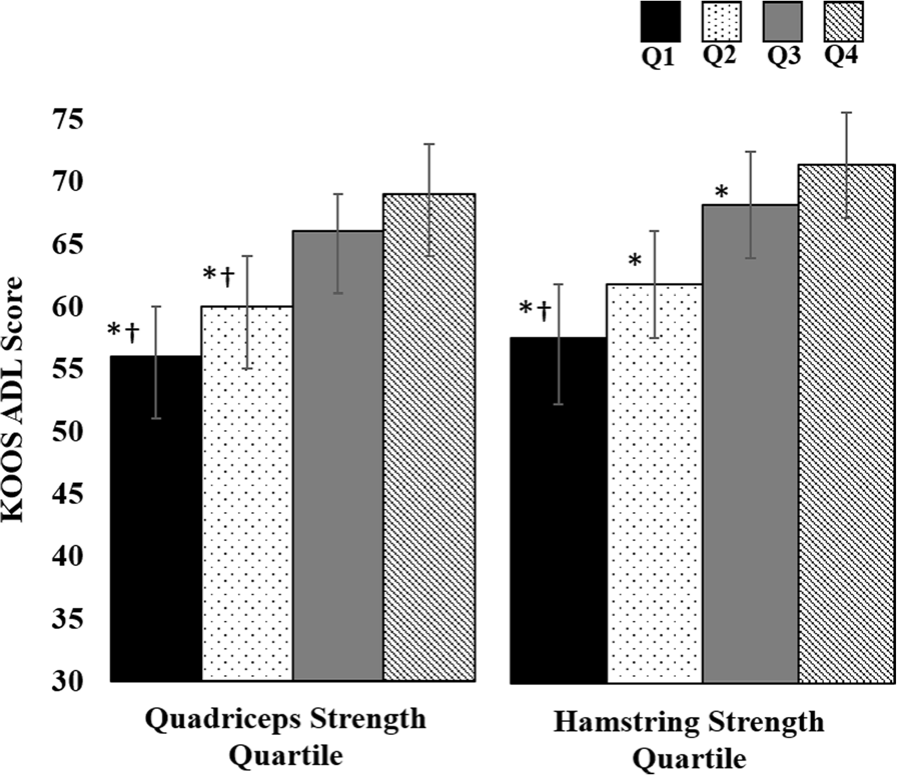
KOOS Activities of Daily Living Scores by Quartile of Muscle Strength. Legend: Data presented as mean Knee injury and Osteoarthritis Outcomes Score (KOOS) activities of daily living score across each quartile (Q) of muscle strength adjusting for structural damage, age, sex and BMI. * indicates significantly less than quartile 4; † indicates significantly less than quartile 3

**Fig. 4:**
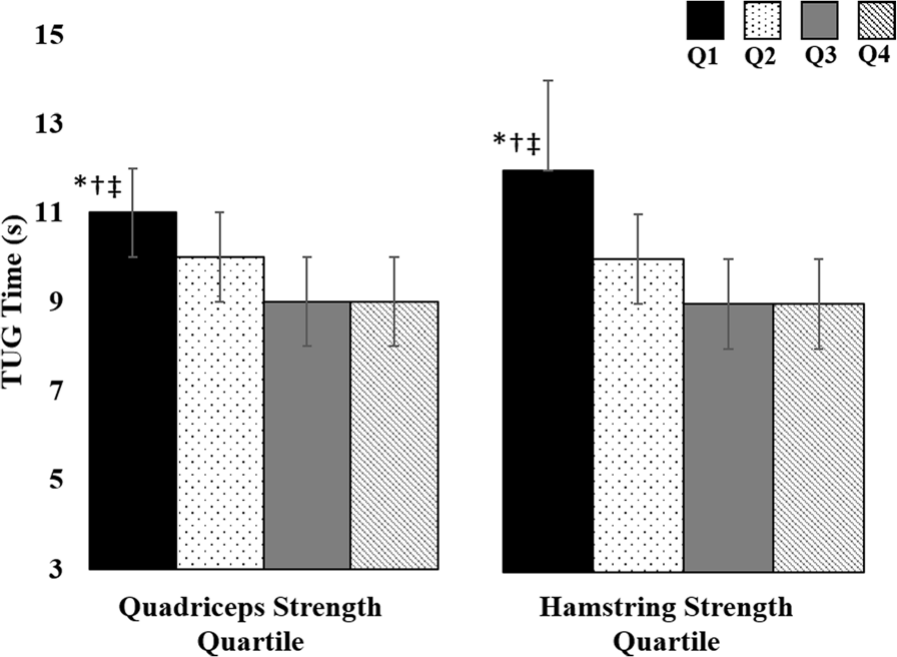
Time to Complete the TUG Test by Quartile of Muscle Strength. Legend: Data presented as mean time to complete the Timed Up and Go (TUG) test across each quartile (Q) of muscle strength adjusting for structural damage, age, sex and BMI. * indicates significantly greater than quartile 4; † indicates significantly greater than quartile 2; ‡ indicates significantly greater than quartile 3

Quadriceps strength was significantly associated with higher KOOS pain scores (mean [SD] Q1 = 44 [16], Q2 = 53 [14], Q3 = 55 [13], Q4 = 62 [15]; *P* = 0.001) and higher KOOS ADL scores (mean [SD] Q1 = 53 [19], Q2 = 60 [17], Q3 = 67 [14], Q4 = 72 [16]; *p* = 0.001). After accounting for age, sex, BMI, and structural damage, the difference in KOOS pain between the strongest and weakest quartiles was clinically meaningful as KOOS pain scores were on average 14 points higher, indicating less pain, in the strongest quartile of quadriceps strength as compared to the weakest quartile (Fig. 2). Similarly, the difference in KOOS ADL scores between the strongest and weakest quartiles was clinically meaningful as KOOS ADL scores were on average 13 points higher, indicating less difficulty performing activities of daily living, in the strongest quartile of quadriceps strength compared to the weakest quartile of quadriceps strength (Fig. 3). Quadriceps strength was associated with less time to complete the TUG test (mean [SD] Q1 = 12 [5], Q2 = 10 [4], Q3 = 9 [3], Q4 = 9 [2]; *p* = 0.009). After accounting for age, sex, BMI, and structural damage, the difference in the time to complete the TUG test between the strongest and weakest quartiles was clinically meaningful as individuals in the strongest quartile of quadriceps strength completed the TUG test an average of 2 s faster than those in the weakest quartile of quadriceps strength (Fig. 4). We did not find a significant association between quadriceps strength and KOOS symptoms scores (mean [SD] Q1 = 43 [15], Q2 = 47 [15], Q3 = 43 [15], Q4 = 47 [13], *p* = 0.226).

### Hamstrings Strength Associations with Structural Damage, Patient-Reported Pain and Functional Status, and Mobility

We did not find that the distribution of K-L grades differed across quartiles of hamstrings strength (Table 1; *p* = 0.36). We did not find that the distribution of the maximum MOAKS cartilage damage size score differed across quartiles of hamstrings strength (Table 1; *p* = 0.54). We did not find that the distribution of the maximum MOAKS cartilage damage depth score was different across hamstrings strength quartiles (Table 1; *p* = 0.84).

Hamstrings strength was associated with higher KOOS pain (mean [SD] Q1 = 46 [16], Q2 = 51 [14], Q3 = 55 [14], Q4 = 63 [14], *p* < 0.001) and higher KOOS ADL scores (mean [SD] Q1 = 55 [18], Q2 = 60 [18], Q3 = 66 [16], Q4 = 74 [14], p < 0.001). After accounting for age, sex, BMI, and structural damage, the differences in KOOS pain and ADL scores between the highest and lowest quartiles were clinically meaningful as KOOS pain scores were on average 13 points higher, indicating less pain, in the strongest quartile of hamstrings strength as compared to the weakest quartile of hamstrings strength (Fig. 2), and KOOS ADL scores were on average 14 points higher, indicating less difficulty performing activities of daily living, in the strongest quartile of hamstrings strength compared to the weakest quartile of hamstrings strength (Fig. 3). Hamstrings strength was associated with less time to complete the TUG test (mean [SD] Q1 = 12 [5], Q2 = 10 [3], Q3 = 9 [3], Q4 = 9 [2], *p* = 0.005). After accounting for age, sex, BMI, and structural damage, the difference in the time to complete the TUG test between the strongest and weakest quartiles was clinically meaningful as individuals in the strongest quartile of hamstrings strength completed the TUG test an average of two seconds faster than those in the weakest quartile of hamstrings strength (Fig. 4). We did not find a significant association between hamstrings strength and KOOS symptoms (mean [SD] Q1 = 44 [15], Q2 = 45 [16], Q3 = 44 [15], Q4 = 47 [14], *p* = 0.925).

## Discussion

In this cross-sectional study of individuals with symptomatic meniscal tear and knee osteoarthritis, we observed that greater quadriceps and hamstrings strength were significantly associated with less patient-reported pain and difficulty completing activities of daily living, and better objectively measured mobility after accounting for age, sex, BMI and structural damage. The difference in pain and difficulty performing activities of daily living between the strongest and weakest quartiles of quadriceps and hamstrings strength exceeded the minimal clinically important difference of 8 points for the KOOS [32]. While our cross-sectional results will need to be tested in longitudinal studies, they suggest that increasing quadriceps and hamstrings muscle strength may be beneficial for reducing pain and difficulty performing activities of daily living, and improving mobility in individuals with symptomatic meniscal tear and knee osteoarthritis.

The quadriceps are critical for generating joint actions and attenuating loading during functional tasks such as walking and rising from a chair [6]. In our study, we determined that quadriceps strength was associated with both subjective and objective clinical features of symptomatic knee osteoarthritis. In general, our results agree with previous studies using laboratory-specific instrumentation to quantify quadriceps muscle strength, as greater strength was associated with better functional task performance and less self-reported disability in individuals with knee osteoarthritis [13–15, 21, 22]. Conversely, other studies have determined weak correlation coefficients between lower extremity muscle strength and pain [34, 35]. Contrasting results between our study and previous investigations are likely due to the evaluation of lower extremity muscle strength during a concentric, bilateral leg extension task in one study [35] or the assessment of knee pain using a visual analog scale [34]. We assessed open-chain quadriceps strength using a hand-held device, which allowed us to isolate the quadriceps muscle on the index limb. Our results suggest that quadriceps strength measured in a clinical setting using a hand-held dynamometer is associated with various clinical features that may contribute to functional limitation in individuals with symptomatic meniscal tear and knee osteoarthritis.

Similar to quadriceps strength, we determined that greater hamstrings strength is associated with less pain, less difficulty performing activities of daily living and greater mobility in individuals with symptomatic meniscal tear and knee osteoarthritis. The hamstrings provide dynamic joint stability during walking [6], and greater patient-reported joint stability has been associated with less knee pain [36]. Our results build upon previous work suggesting that hamstrings strength contributes to both subjective [19] and objective [14, 15] clinical features of knee osteoarthritis. Previous studies have determined greater hamstrings strength is associated with less pain and better performance on stair climb and sit to stand assessments [14, 15, 19]. Our results suggest that hamstrings strength contributes to multiple activities of daily living as greater hamstrings strength associated with time to complete the TUG test, which comprises both balance and mobility, and KOOS ADL scores, which assesses the difficulty experienced while completing a variety of daily activities ranging from sitting to ascending and descending stairs. Our results suggest that multiple lower extremity muscles contribute to clinical features of knee osteoarthritis, and intervention strategies aiming to improve both quadriceps and hamstrings strength may be beneficial.

Classifying our participants into quartiles of quadriceps and hamstrings strength allows us to determine the clinical relevance of quadriceps and hamstrings muscle strength regarding subjective and objective clinical features of knee osteoarthritis. In our cohort, the difference in mean KOOS pain and KOOS ADL scores between the strongest and weakest quartiles of quadriceps and hamstrings muscle strength when adjusted for structural damage and demographic characteristics ranged from 13 to 15 points (Figs. 1 and 2), which represents a clinically meaningful difference in KOOS scores [32]. While we cannot determine how changes in quadriceps and hamstrings muscle strength are associated with changes in clinical features, a previous study has determined that a small reduction in quadriceps and hamstrings muscle strength of approximately 6% was associated with a clinically relevant decline in physical function quantified via the WOMAC [12]. Similarly, individuals in the weakest quartiles of quadriceps and hamstrings muscle strength in our study took on average 2 to 3 s longer to complete the TUG test compared to those in the strongest quartiles, which is greater than the minimum detectible difference of 1.14 s [33]. The TUG test assesses balance and self-selected walking speed, and a greater time to complete the TUG test has been associated with fall risk [37]. Additionally, individuals with slower walking speed are also less likely to meet physical activity guidelines [4]. Individuals with or at risk of developing knee osteoarthritis who take more steps per day have a lower risk of developing functional limitation over 2 years compared to those who take fewer steps per day [3]. Future research is needed to determine how quadriceps and hamstrings muscle strength may contribute to additional features of knee osteoarthritis, such as decreased physical activity, and how improving physical activity may be beneficial for patients with symptomatic meniscal tear and knee OA.

In recent years, multiple clinical trials have demonstrated that arthroscopic partial meniscectomy offers little additional improvement in pain when compared to non-operative interventions for the treatment of symptomatic meniscal tear [26, 38–40]. As the management of symptomatic meniscal tear and knee osteoarthritis transitions to conservative approaches, our results highlight the importance of appropriately strengthening the quadriceps and hamstrings musculature. Previous work demonstrated that a 12-week neuromuscular and strength exercise program consisting of squats, lunges, leg press and hamstring curl exercises improved quadriceps and hamstring muscle strength in patients with meniscal tear [41]. Additionally, high intensity strength training and high velocity power training have been shown to be effective at increasing quadriceps and hamstrings muscle strength in individuals with knee osteoarthritis [42]. Utilizing high intensity strength training and power training may be beneficial for improving lower extremity muscle strength in patients with symptomatic meniscal tear and knee osteoarthritis, and therefore may improve pain and physical function. However, gains in muscle strength following training programs may be limited by underlying deficits in voluntary activation [22, 43]. While improving quadriceps and hamstrings muscle strength is likely beneficial for improving clinical features of knee osteoarthritis, rehabilitation programs may need to treat underlying neural factors (i.e. arthrogenic muscle inhibition) that limit the ability to fully restore muscle strength. Future research is needed to determine the most efficacious therapeutic interventions for improving lower extremity muscle strength in patients with symptomatic meniscal tear and knee osteoarthritis.

While this study improves our understanding of the association between quadriceps and hamstrings muscle strength and subjective and objective clinical features of knee osteoarthritis, there are limitations that should be addressed to inform future research. As our study is cross-sectional in nature, we are unable to determine the causal relationship between quadriceps and hamstrings muscle strength and clinical features of knee osteoarthritis. We also cannot determine how other lower extremity muscles, including the gluteus medius and gluteus maximus, may have contributed to KOOS scores and time to complete the TUG in our study. We limited our inclusion criteria to individuals with mild to moderate knee osteoarthritis based upon K-L grade, therefore it is unknown how our results translate into the larger population of individuals who are at risk of developing knee osteoarthritis or those with end-stage disease. Additionally, we did not determine the presence of bilateral osteoarthritis in this cohort, therefore it remains unknown if the presence of bilateral osteoarthritis may influence our results. We used a hand-held dynamometer to assess quadriceps and hamstrings muscle strength due to its cost-effectiveness, portability and ease of use, rather than an isokinetic dynamometer. However, strong, positive associations (i.e., Pearson correlation coefficient range = 0.72–0.85) between the assessment of quadriceps and hamstrings muscle strength using a hand-held dynamometer and an isokinetic dynamometer have been reported [23]. We also used inelastic straps to stabilize the dynamometer and maintain joint positioning during the assessments, which has been demonstrated to improve the reliability of these measures [30]. We chose to normalized peak force in newtons to body mass to account for differences in body size. The lever arm length was not available in this cohort; therefore, we are unable to determine joint torque. Previous research, however has demonstrated that taking the lever arm length into account for the purpose of normalizing muscle strength does not influence results when compared to normalizing peak force to body mass [19]. Lastly, there are additional factors that may influence quadriceps and hamstrings muscle strength, including neuromuscular activation, muscle co-contraction, and joint range of motion, and we are unable to discern how additional unmeasured factors may contribute to quadriceps and hamstrings muscle strength.

## Conclusions

In conclusion, our results demonstrate that quadriceps and hamstrings muscle strength, assessed using a hand-held dynamometer, was associated with subjective and objective clinical features in individuals with symptomatic meniscal tear and knee osteoarthritis. Individuals classified into the strongest quartiles of quadriceps and hamstrings strength reported less pain, less difficulty performing activities of daily living, and demonstrated better mobility than those in the weakest quartiles. The differences in pain and difficulty performing activities of daily living between the strongest and weakest quartiles of muscle strength exceeded the clinically meaningful difference for the KOOS. These results should be confirmed in trials or longitudinal studies, but they suggest that that quadriceps and hamstrings muscle strengthening may be a key component for the non-operative management of symptomatic meniscal tear and knee osteoarthritis.

## Abbreviations

ADL: Activities of Daily Living
BMI: Body Mass Index
K-L: Kellgren-Lawrence
KOOS: Knee Injury and Osteoarthritis Outcomes Score
MeTeOR: Meniscal Tear in Osteoarthritis Research
MOAKS: MRI OA Knee Score
N: Newtons
Q: Quartiles
TUG: Timed Up and Go Test
